# NETS^1HD^: study protocol for development of a core outcome set for use in determining the overall success of Hirschsprung’s disease treatment

**DOI:** 10.1186/s13063-016-1693-6

**Published:** 2016-12-07

**Authors:** Benjamin Allin, Timothy Bradnock, Simon Kenny, Gregor Walker, Marian Knight

**Affiliations:** 1 National Perinatal Epidemiology Unit, University of Oxford, Old Road Campus, Richard Doll Building, Oxford, OX3 7LF UK; 2 Oxford University Hospitals NHS Trust, Headley Way, Headington, Oxford, OX3 9DU UK; 3 Royal Hospital for Children, Glasgow, 1345 Govan Road, Govan, Glasgow, G51 4TF UK; 4 Alder Hey Children’s Hospital, East Prescot Road, Liverpool, L14 5AB UK

**Keywords:** Paediatric surgery, Hirschsprung’s disease, Core outcome sets

## Abstract

**Background:**

Use of core outcome sets in research has been proposed as a method for countering the problems caused by heterogeneity of outcome measure reporting. Heterogeneity of outcome measure reporting occurs in Hirschsprung’s disease (HD) research and is limiting the development of a robust evidence base to support clinical practice.

**Methods:**

Candidate outcome measures have been identified through a systematic review. These outcome measures will form the starting point for a three-phase online Delphi process to be carried out in parallel by three panels of experts. Panel 1 is a neonatal panel; panel 2 is a non-neonatal panel; and panel 3 is a lay panel. In round 1, experts will be asked to score the previously identified outcome measures from 1 to 9 based on how important they think the measures are in determining the overall success of their/their child’s/their patient’s HD. In round 2, experts will be presented with the same list of outcome measures and graphical representations of how their panel scored that outcome in round 1. They will be asked to re-score the outcome measure, taking into account how important other members of their panel felt it to be. In round 3, experts will again be asked to re-score each outcome measure, but this time they will receive a graphical representation of the distribution of scores from all three panels, which they should take into account when re-scoring. Following round 3 of the Delphi process, 40 experts will be invited to attend a face-to-face consensus meeting. Participants will be invited in a purposive manner to obtain balance between the different panels. Results of the Delphi process will be discussed, and outcomes will be re-scored. Outcome measures where >70% of participants at the meeting scored it 7–9 and <15% scored it 1–3 will form the core outcome set.

**Discussion:**

Development of a core outcome set will help to reduce heterogeneity of outcome measure reporting in HD. This will increase the quality of research taking place and ultimately improve care provided to infants with HD.

**Electronic supplementary material:**

The online version of this article (doi:10.1186/s13063-016-1693-6) contains supplementary material, which is available to authorized users.

## Background

Hirschsprung’s disease (HD) is a condition where the nerves of the myenteric plexus do not develop correctly along the full length of the colon. It results in infants being unable to open their bowels properly, and it affects approximately 1.7 in 10,000 live births in the United Kingdom and Ireland. Treatment is usually with surgical resection of the affected sections of bowel within the first few months of life. There are currently three main approaches to conducting the definitive pull-through procedure required for treatment of HD: the open approach, the purely transanal approach [1] and the laparoscopically assisted approach [2]. There are also multiple methods used to perform colorectal/coloanal anastomosis [3–5]. To date, authors of systematic reviews have been unable to reliably determine the gold standard intervention for HD [6, 7]. One of the key factors preventing identification of a gold standard intervention is heterogeneity of outcome measure reporting in primary studies.

Heterogeneity of outcome measure reporting results in difficulties with synthesising data from individual studies, as well as resulting in studies being prone to reporting bias and a lack of patient relevance [8]. Within HD research, such heterogeneity has resulted in the conduct of studies focused on hospital-based measures or surrogate markers of success, as opposed to outcomes that are deemed important by people with HD or their families [7]. Owing to the low incidence of HD, conducted studies are frequently limited in size. In this situation, it is essential that all conducted studies be relevant to patients, report full data and be easy to synthesise with other work.

A core outcome set (COS) is a group of outcome measures that have been identified by key stakeholder groups as the most important in determining the success of treatment of a particular condition [8, 9]. Use of a COS to define the outcome measures which are the minimum it is acceptable to investigate and report in a particular field of research has been shown to be a successful method for countering heterogeneity in outcome measure reporting [10]. When development of a COS for HD is combined with increased collaboration between research institutions, the ability of HD research to answer clinically relevant questions will be increased, and generation of robust evidence-based management guidelines will be facilitated.

## Methods

### Ethics and registration

The Health Research Authority deemed the project to be service evaluation/service development, and therefore review by a National Health Service (NHS) Research Ethics Committee was not deemed necessary. Information on the nature of the study will be provided to participants prior to registration and again prior to completion of the first round of the survey. Potential participants are given contact details for staff within the National Perinatal Epidemiology Unit (NPEU) from whom further information can be obtained or with whom they can discuss the study further. Detailed information is also available on the study website (http://www.npeu.ox.ac.uk/nets). Consent to participate in the study is implied by completion of the registration questionnaire and data collection questionnaires. Participants can withdraw from the study at any time, either by contacting the study team or by simply not completing a data collection questionnaire. The study has been registered with the Core Outcome Measures in Effectiveness Trials (COMET) Initiative (http://www.comet-initiative.org).

### Scope of the core outcome set

The developed COS is intended to be used to assess the overall success of treatment of an infant born with HD. This will involve outcome measures identified as important from diagnosis through into adulthood. We have kept the scope of the COS broad for two reasons. First, although HD research currently covers multiple different areas of treatment, there are only a limited number of studies being conducted that use robust methodology [11]. Any intervention that is aimed at improving the overall robustness of research taking place therefore needs to be as broadly applicable as possible. Second, whilst research into the treatment of infants with HD will fall into three broad areas (management of enterocolitis, definitive surgery and re-do surgery), it is the combination of success of treatment in these three areas that matters. Developing a COS to reflect treatment success in one area whilst neglecting the importance of the other two areas would therefore likely not meet the primary aim of developing the COS.

The following are key objectives of the trial:

1. To determine which outcomes are currently reported in studies comparing surgical treatments for HD and assess the quality of reporting
2. To prioritise outcomes from patient/parent, paediatric surgical and non-surgical clinician perspectives
3. To achieve consensus between key stakeholders on a COS for assessing how successful the overall treatment of an infant with HD has been
4. To compare and contrast outcomes prioritised by patients/parents, surgeons and non-surgical clinicians

Wider aims of the trial are as follows:

1. To develop methodology for addressing difficulties likely to be encountered in development of COSs in all paediatric surgical conditions, including the following:

- Recruitment of parents and patients
- Incorporation of opinions from clinicians whose priorities vary dependent on their specialty, or age they encounter the child

### Design

There will be four key stages to development of the COS:

1. Systematic review to identify currently reported outcomes
2. Development of a panel of experts
3. Three-phase online Delphi process
4. Consensus meeting

### Systematic review

An already conducted systematic review has been used as the basis for phase 1 of the Delphi process (Allin B, Irvine A, Patni N, Knight M. Variability of outcome reporting in Hirschsprung’s disease and gastroschisis: a systematic review, unpublished). The aim of this systematic review was to identify every outcome measure investigated by studies that have been conducted since 2010 and involved more than ten participants, and in which two or more interventions for HD were compared. The systematic review included both observational and interventional studies and thereby gave a broad overview of outcomes that are currently investigated in the HD literature. The outcome domains identified by this systematic review will be used as the outcomes to be scored in phase 1 of the Delphi process. Specific time points and methods of measurement will not be incorporated into the outcomes to be scored in phase 1. These components will instead be identified through subsequent literature review; discussion at the consensus meeting; and, if necessary, a separate ‘how to measure’ meeting. The use of systematic reviews to inform phase 1 of a Delphi process is recommended by the COMET Initiative [12] and has previously been used in the development of other COSs, including in the Management of Otitis Media with Effusion in Cleft Palate study [13].

### Panel assembly

#### Expert identification and recruitment

To ensure appropriate breadth of recruitment, experts will be recruited under three broad categories (disciplines, organisations and literature) and then within sub-categories, which include, amongst others, paediatric surgeons, paediatric gastroenterologists, parents, the Royal College of Paediatrics and Child Health, and the *Journal of Paediatric Surgery*. Members of the study management group (SMG) will draw up an exhaustive list of sub-categories from which at least two participants must be identified. Experts known to the SMG and fitting into one of these sub-categories will be identified first, prior to expanding recruitment using methods specific to each sub-category. Examples of expert recruitment include the following:

- Contacting all paediatric surgeons listed on the British Association of Paediatric Surgeons register of practitioners as having a specialist interest in the treatment of infants with HD
- Advertising to people with HD and their parents via mailing lists established by the Hirschsprung’s & Motility Disorders Support Network
- Contacting the editors of key paediatric surgical journals and asking them to identify members of their editorial board with an interest in HD
- Advertising to paediatric gastroenterologists via mailing lists of the British Society of Paediatric Gastroenterology Hepatology and Nutrition

All identified experts will be emailed a link to an online form on which they can detail their involvement in HD and express their interest in participating in the study. Experts will also be asked to provide the names of anyone else they feel would be appropriate to invite to participate in the study. At this stage, we are seeking to clarify the suitability of experts for participation in the study and to identify further potential experts. This process will be repeated for the names provided by already-contacted experts. Expert recruitment will continue until a minimum of 50 experts, at least 2 in each category, have been recruited.

Following confirmation of their eligibility to participate in the study, experts will be sent a link to a customised online database hosted on the secure servers of the University of Oxford and developed using LimeSurvey (https://www.limesurvey.org), from which they can access phase 1 of the Delphi process.

#### Facilitating consensus

To be truly representative of the broad experience of those involved in HD, it is important that the COS reflect the opinions of people with HD, their parents, and a broad spectrum of clinicians, including those who treat people with HD later in life (e.g., paediatric gastroenterologists), as well as in early infancy (e.g., neonatologists). These different groups may have different priorities, which could cause difficulties in attaining consensus on a single set of important outcome measures. Thus, to facilitate achieving consensus, experts will be separated into three different panels:

1. *Neonatal panel*: Clinicians whose responsibility includes management in the neonatal period (but may also include management outside the neonatal period). This group will include neonatologists and paediatric surgeons.
2. *Non-neonatal panel*: Researchers with expertise in HD management and clinicians responsible for management primarily outside the neonatal period. This group will include specialist nurses and paediatricians.
3. *Lay panel*: Parents (or other equivalent guardian) and adults born with HD.

### Delphi process

#### Phase 1 data collection

To maintain anonymity and ease data collection, a customised online system will be developed to conduct a three-phase Delphi process run in parallel for the neonatal, non-neonatal and lay panels. In phase 1, participants will be presented, in alphabetical order, with outcomes identified from the systematic review. Lay equivalents will be developed for each outcome measure, and these will be used instead of scientific terms for the parent/patient panel. These terms will be developed in conjunction with the NPEU’s parent advisory group.

Participants will be asked to give each outcome measure a score from 1 to 9, where 1, 2 and 3 are ‘not that important’; 4, 5 and 6 are ‘important’; and 7, 8 and 9 are ‘really important’. The Grading of Recommendations Assessment, Development and Evaluation (GRADE) scale of measurement has been chosen for use in scoring outcome measures on the basis of recommendations from the COMET Initiative [12]. After scoring all outcome measures identified by the systematic review, participants will be asked to list any additional outcome measures they feel are important in determining the success of treatment of HD, but which we have not already asked them to score. These outcomes will not be scored in phase 1.

Data will be collected over a 4-week period for each phase of the Delphi process. Participants who have not completed the survey will be sent reminders via email when they have 2 weeks, 1 week and 48 h remaining for completion of the survey. Participants who have not completed the questionnaire within 4 weeks of the phase start will be deemed not to have completed that phase. Testing has suggested that completion of the phase 1 questionnaire will take participants approximately 15 minutes.

#### Phase 1 analysis

The number of experts invited to participate, registering to participate and completing phase 1 of the Delphi process from each sub-category will be recorded. Outcomes will be analysed separately for each panel, with descriptive statistics calculated, including medians and inter-quartile ranges. All outcomes will be carried forward to phase 2. Additional outcomes listed by experts in phase 1 will be assessed by the SMG to determine whether they represent de novo outcomes and whether they fall within the scope of the COS. Those that are deemed to both fall within the scope of the COS and represent a de novo outcome will be taken forward to phase 2 and scored alongside the outcome measures scored in phase 1. We anticipate that there will be between 10 and 20 outcomes that meet these criteria.

#### Phase 2 data collection

Experts completing phase 1 will be invited to participate in phase 2 and asked to re-score each outcome on the basis of the following:

- The phase 1 score they assigned it
- Descriptive statistics from their panel

Descriptive statistics will be represented numerically and graphically.

#### Phase 2 analysis

Descriptive statistics will again be calculated. Bias from loss of experts between rounds will be assessed by determining if there is any difference in median round 1 scores for each outcome measure between experts who have completed both phases and experts who completed only phase 1. All outcomes will be carried forward to phase 3.

#### Phase 3 data collection

Experts completing phase 2 will be invited to participate in phase 3 and asked to re-score each outcome on the basis of the following:

- The phase 2 score they assigned it
- Round 2 descriptive statistics from all three panels

#### Phase 3 analysis

Analysis will be conducted as per phase 2.

### Generation of core outcome set: consensus meeting

A roundtable consensus meeting with an independent, non-voting chair will be held at a central location in the United Kingdom to identify the final COS and determine how to measure those outcomes that form it. Participants will be invited to attend the consensus meeting in such a way as to ensure participation from all three panels that is as even as possible, with a range of experiences represented.

The final COS will comprise those outcome measures where >70% of participants at the consensus meeting score the outcome measure between 7 and 9 and less than 15% of participants at the consensus meeting score the outcome measure between 1 and 3. Every outcome measure assessed in phase 3 of the Delphi process will be discussed and re-scored by meeting participants using the same scale as for the online portion of the study.

**Fig. 1:**
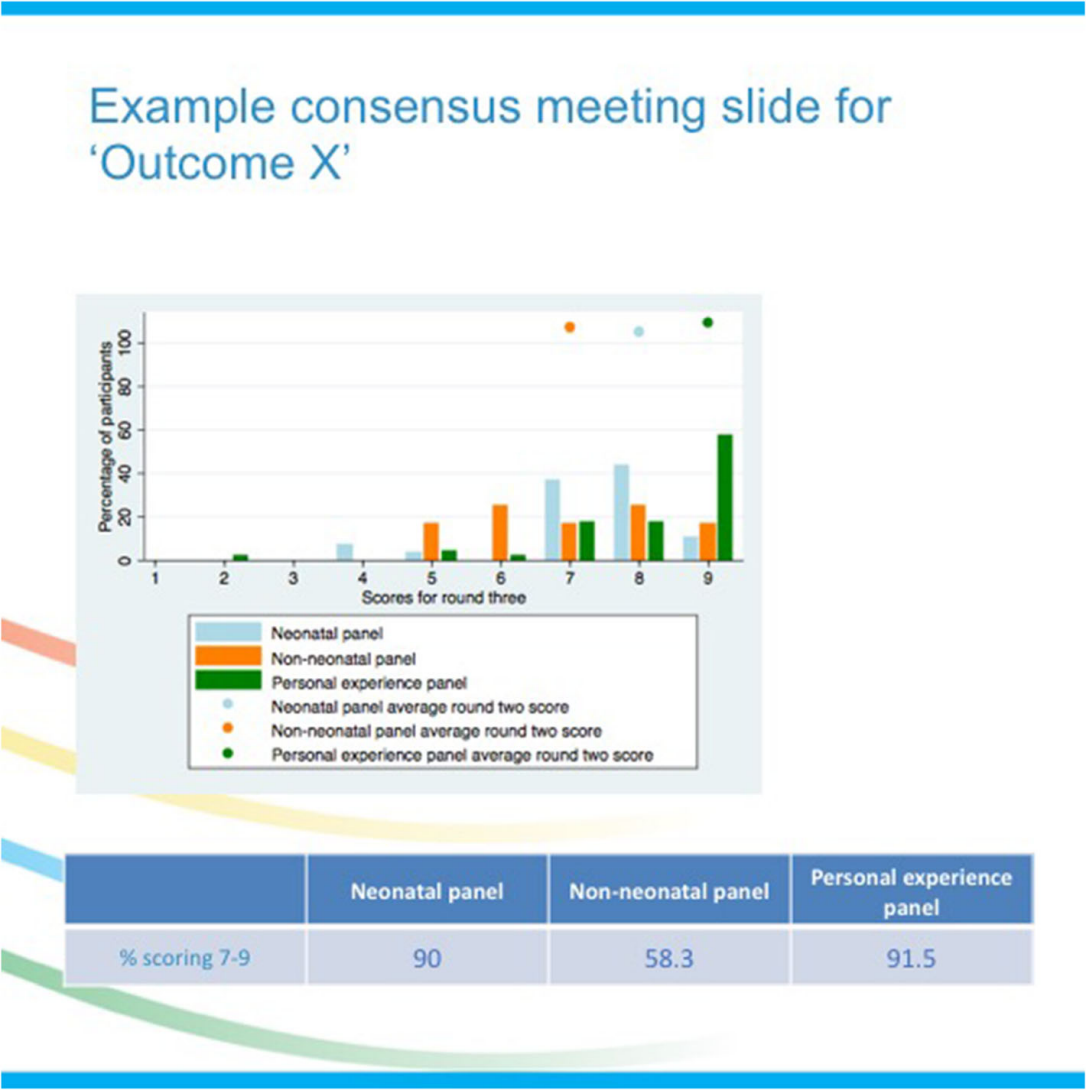
Sample consensus meeting slide

Prior to the meeting, participants will be given a summary of the final results of the Delphi process. During the meeting, they will be shown a graphical representation of the scores for each outcome measure (Fig. 1) and given the opportunity to explore the reasons for any differences in scores between panels. After discussion of each outcome measure, re-scoring will take place electronically and anonymously using the TurningPoint system (Turning Technologies, Youngstown, OH, USA). These scores will be used to determine whether an outcome measure is included in the COS.

Following completion of the consensus meeting, a consensus document will be drafted and put forward to participants for approval. This document will be presented at appropriate international meetings and published in peer-reviewed journals.

### Data management

All data will be directly entered into a customised database by participants. Data will be stored securely on servers within the NPEU and will be managed as per standard operating protocols. Only appropriate members of the study team will have access to the data. Data analysis will be conducted by BA and MK. Standard Protocol Items: Recommendations for Interventional Trials (SPIRIT) checklists are provided in Additional file 1.

## Discussion

Development of the COS will identify outcomes that are important to people with HD, their parents and key clinical stakeholder groups. Use of such a COS in future research will help to counter many of the previously identified problems caused by heterogeneity of outcome measure reporting, including a lack of patient relevance, a high risk of reporting bias and difficulty in conducting meta-analyses.

Involvement of multiple stakeholder groups in development of the COS is one of its major strengths and will ensure that it is relevant to patients and applicable across a broad range of clinical settings. This is the first time that people with HD and their parents will have been involved in identification of key outcomes for research studies in a robust, replicable manner, and this effort will help to ensure that future research has a genuine patient-centred focus.

Although the COS will seek to involve participants from a range of countries involved in the management of infants with HD, none will be recruited from low- or middle-income countries. This is because it is felt likely that priorities in those countries will be very different from those in high-income countries, and therefore inclusion of participants from those settings would create too heterogeneous a study population. Given the lack of involvement of these countries and their likely difference in priorities, the most significant limitation of this COS is therefore that it is likely to be valid only for use in high-income countries.

Despite the likely limitation of its applicability to high-income countries, we believe the developed COS will have application across a wide range of HD research. One of the major challenges associated with development of the COS, however, will be ensuring that its use is taken up by other research groups. To ensure that this occurs, key paediatric surgical stakeholders, including the British Association of Paediatric Surgeons, are involved in development of the COS and will endorse the study to their memberships. Funding has also been obtained to conduct a UK-wide cohort study to investigate the identified core outcomes in infants with HD as they turn 6 years of age. Implementing the COS in a study with the power to address key clinical questions will help to demonstrate that its use is both feasible and desirable.

Use of COSs in other specialties has been shown to improve the quality of research taking place [10]. This will be the first time a COS has been developed for use in a paediatric surgical condition, and we hope it will create the infrastructure and methodology required to replicate the process in other conditions, leading to a generalised increase in the robustness of the evidence base used to support clinical practice in paediatric surgery.

## Trial status

Data collection finished after submission of the manusript, but prior to publication.

## Additional file

Additional file 1: SPIRIT checklist detailing where key sections of text can be identified within the protocol. (DOC 126 kb)

## Abbreviations

COMET: Core Outcome Measures in Effectiveness Trials
COS: Core outcome set
GRADE: Grading of Recommendations Assessment, Development and Evaluation
NHS: National Health Service
NIHR: National Institute for Health Research
NPEU: National Perinatal Epidemiology Unit
SMG: Study management group
SPIRIT: Standard Protocol Items: Recommendations for Interventional Trials
